# Smartphone Apps for the Treatment of Mental Disorders: Systematic Review

**DOI:** 10.2196/14897

**Published:** 2020-04-02

**Authors:** Ignacio Miralles, Carlos Granell, Laura Díaz-Sanahuja, William Van Woensel, Juana Bretón-López, Adriana Mira, Diana Castilla, Sven Casteleyn

**Affiliations:** 1 Universitat Jaume I Castellón de la Plana Spain; 2 Dalhousie University Halifax, NS Canada; 3 CIBER of Physiopathology of Obesity and Nutrition CIBERobn Castellón Spain; 4 Department of Personality, Evaluation and Psychological Treatment University of Valencia Valencia Spain

**Keywords:** mental health, mental disorders, treatment, intervention, mHealth, smartphone, mobile phone, mobile apps, systematic review

## Abstract

**Background:**

Smartphone apps are an increasingly popular means for delivering psychological interventions to patients suffering from a mental disorder. In line with this popularity, there is a need to analyze and summarize the state of the art, both from a psychological and technical perspective.

**Objective:**

This study aimed to systematically review the literature on the use of smartphones for psychological interventions. Our systematic review has the following objectives: (1) analyze the coverage of mental disorders in research articles per year; (2) study the types of assessment in research articles per mental disorder per year; (3) map the use of advanced technical features, such as sensors, and novel software features, such as personalization and social media, per mental disorder; (4) provide an overview of smartphone apps per mental disorder; and (5) provide an overview of the key characteristics of empirical assessments with rigorous designs (ie, randomized controlled trials [RCTs]).

**Methods:**

The Preferred Reporting Items for Systematic Reviews and Meta-Analyses guidelines for systematic reviews were followed. We performed searches in Scopus, Web of Science, American Psychological Association PsycNET, and Medical Literature Analysis and Retrieval System Online, covering a period of 6 years (2013-2018). We included papers that described the use of smartphone apps to deliver psychological interventions for known mental disorders. We formed multidisciplinary teams, comprising experts in psychology and computer science, to select and classify articles based on psychological and technical features.

**Results:**

We found 158 articles that met the inclusion criteria. We observed an increasing interest in smartphone-based interventions over time. Most research targeted disorders with high prevalence, that is, depressive (31/158,19.6%) and anxiety disorders (18/158, 11.4%). Of the total, 72.7% (115/158) of the papers focused on six mental disorders: depression, anxiety, trauma and stressor-related, substance-related and addiction, schizophrenia spectrum, and other psychotic disorders, or a combination of disorders. More than half of known mental disorders were not or very scarcely (<3%) represented. An increasing number of studies were dedicated to assessing clinical effects, but RCTs were still a minority (25/158, 15.8%). From a technical viewpoint, interventions were leveraging the improved modalities (screen and sound) and interactivity of smartphones but only sparingly leveraged their truly novel capabilities, such as sensors, alternative delivery paradigms, and analytical methods.

**Conclusions:**

There is a need for designing interventions for the full breadth of mental disorders, rather than primarily focusing on most prevalent disorders. We further contend that an increasingly systematic focus, that is, involving RCTs, is needed to improve the robustness and trustworthiness of assessments. Regarding technical aspects, we argue that further exploration and innovative use of the novel capabilities of smartphones are needed to fully realize their potential for the treatment of mental health disorders.

## Introduction

### Background

The popularity of smartphones has skyrocketed over the last decade. Different sources estimate that around 35% of people worldwide own a smartphone; even much higher penetration rates are reported in developed countries (ranging from 76% in the United Kingdom to 95% in South Korea) [1,2]. Smartphones are fast becoming the most common mobile phone, even in emerging economies [2]. Smartphones distinguish themselves from other types of mobile phones in several ways: (1) improved modality (screen and sound), interaction, and computational resources, which support sophisticated software applications called (mobile) apps; (2) built-in mobile sensors, which allow apps to access various measurements, such as the user’s current position, motion, ambient light, and sound; and (3) connectivity hardware (Wi-Fi and Bluetooth), which allows virtually ubiquitous internet connections, as well as connections to nearby wireless hardware (eg, headsets and physiological sensors). A variety of mobile apps have been developed, conveniently installable from so-called app stores, which address a wide range of personal, entertainment, and business needs. In 2017, 2.8 and 2.2 million apps were available from the Google Play and Apple App stores, respectively; collectively, these were downloaded a total of 178.1 billion times [3].

Researchers quickly realized the potential of mobile apps in health [4] and mental health [5], with systematic reviews on related research, that is, targeting mobile health (mHealth) apps, appearing as early as 2011 [5]. Whereas traditional telehealth [6] and cellphone-based [7] systems are limited to SMS, telephone, or video calls, smartphones present a more versatile, powerful, and personalized platform for a holistic set of care tasks, including patient screening, symptom and disorder assessment, psychoeducation, intervention delivery, progress monitoring, and relapse prevention [5]. By providing these health tasks via smartphone apps, albeit partially or combined with a therapist’s intervention, a number of obstacles for mental health care are reduced such as therapist workload, lack of qualified personnel, geographic barriers, and attitudinal barriers to seek treatment. New opportunities arise as well, such as improving assessment by leveraging built-in smartphone sensors (eg, biofeedback and motion) and analyzing device usage, and providing ecological interventions directly to the patient when they are most needed, as determined by in situ assessments [8-14].

This is a timely evolution, as reported mental health problems are becoming increasingly prevalent. Trautmann et al [15] estimated that over 50% of the population of high- and middle-income countries suffers from at least one mental disorder in their life, with a significant impact on their quality of life and an overall annual economic cost of US $2.5 trillion (2010) and rising. According to the latest US annual survey [16], there is an estimated 12-month mental disorder prevalence of 18.3% among adults (4.2% for serious mental illness). Mobile mental health interventions have reported promising mental health outcomes [17-19], large acceptance rates by patients [20], and increased sustainability and preservation of treatment effects [21]. Hence, owing to their ability to reduce obstacles for mental health care, these interventions can be leveraged to meet present-day mental health challenges. Nevertheless, we find that the possibilities of current smartphone technology have only just been tapped, and further research is needed to explore them fully [22], as are studies to rigorously analyze the empirical effectiveness of these systems [22,23]. For driving and steering such future research, there is a continuous need to establish a state of the art, which comprehensively reviews current focal points on psychological (ie, type of disorder and evaluation) and technological factors (smartphone capabilities, technologies, and features used). Such a review should include both exploratory research, which investigates technological opportunities, and empirical research, which establishes robust empirical evidence for the efficacy of smartphone interventions. Previous mobile mental health reviews have become dated [24-26], while more recent studies only consider specific mental disorders; for example, cognitive impairment [27], alcohol and substance abuse [28], anxiety [17]; only consider technologies, for example, text messaging [29] and SMS messages [11]; or focus solely on efficacy, usability, and feasibility of interventions realized by mHealth (mobile health, referring to the use of mobile computing and communication technologies in health care [29]) interventions [11,17,27,30,31].

### Objectives

We provide a systematic review that studies the recent (2013-2018) research on smartphone app-based interventions for mental disorders. Specifically, we aimed to analyze and summarize relevant research to (1) analyze the coverage of mental disorders in research articles per year; (2) study the types of assessment in research articles per mental disorder per year; (3) map the use of sensors, software features, and analytical capabilities of smartphones per mental disorder; (4) provide an overview of mobile smartphone apps per mental disorder; and (5) provide an overview of the key characteristics from empirical assessments with rigorous designs (ie, randomized controlled trials [RCTs]). As a counterbalance to our focus on smartphone interventions, we also briefly discuss potential risks such as lack of proven effectiveness, possibility for harm, and breach of privacy.

## Methods

### Search and Study Selection

This systematic review uses the Preferred Reporting Items for Systematic Reviews and Meta-Analyses (PRISMA) [32] as a guideline. We performed an extensive search of scientific databases, that is, Scopus, Web of Science (WoS), American Psychological Association (APA) PsycNET, and Medical Literature Analysis and Retrieval System Online (MEDLINE), using queries that combined search terms related to the psychological (eg, psychology, psychological, mental disorder and intervention) and the technological dimensions (eg, mobile device, smartphone and mHealth) using logical operators. All database-specific queries were semantically equivalent but formulated using the different syntaxes and technical support of the respective search engines. The queries were launched on March 9, 2018, covering results from 2013 until March 2018, and relaunched on July 13, 2019, to cover the full year of 2018. Keywords and queries can be found in Multimedia Appendices 1 and 2.

All resulting publications were downloaded, and duplicates were removed. All papers were equally divided among four multidisciplinary groups of two members, each comprising one computer scientist and one psychologist. Publications were initially screened based on the inclusion/exclusion criteria (IC/EC), using title, abstract, and keywords. Subsequently, papers that were still inconclusive, that is, after initially screening for their title, abstract, and keywords, were fully reviewed to check their eligibility using the IC/EC. Both during initial screening and full-text screening for eligibility, both team members processed the group’s assigned papers independently and discussed their observations before making a final decision. In case of disagreement, a third reviewer was assigned, and a final decision was made collaboratively.

### Inclusion Criteria

Articles fulfilling all the following IC were included in our systematic review: (*IC1*) Full research articles published in an international journal or conference proceedings between January 1, 2013, and December 31, 2018, written in English, and where a full text was available; (*IC2*) Primary research articles, that is, articles that produce first-hand contributions to the research field; (*IC3*) Articles explicitly describing the use of a smartphone app for the delivery of psychological intervention(s) for mental disorder(s), whereby (a) smartphones are used as delivery platform and at least one smartphone-specific feature is used, thus going beyond regular mobile phone features (eg, SMS messages and phone calls) and standard content delivery (eg, nonmobile and generic websites); (b) the targeted mental disorders are found in the Diagnostic and Statistical manual of Mental Disorders (DSM-5) [33]; and (*IC4*) Articles including either exploratory research (ie, investigating technological opportunities) or empirical research (ie, establishing robust empirical evidence). For *exploratory research*, an explicit description on the use of the smartphone app for a psychological intervention is required. For *empirical research*, there were no restrictions on study design. Study protocols were also included.

### Exclusions Criteria

EC were all sources that do not comply with the IC: (*EC1*) All research articles published before 2013 or after 2018, not written in English, not published as a full paper in an international journal or conference. This excludes articles published in any other outlet, such as workshops, discussion forums, colloquia, patent descriptions, white papers, and other types of publications, for example, posters, demo papers, tutorial paper, editorials, or extended abstracts; (*EC2*) All secondary research articles, that is, articles that use primary research articles to derive results such as reviews, systematic maps, meta-analysis, synthesis, and comments; (*EC3*) Any article not explicitly describing the use of smartphones as the primary mode of delivering psychological interventions for mental disorders. This excludes articles addressing nonmental disorders (eg, cancer) or symptoms (eg, stress), as well as articles describing the use of other mobile devices (eg, wearables, smart watches, and tablets) or using smartphones only as a regular phone (eg, SMS messages and phone calls); and (*EC4*) Any article that only superficially describes the application of a smartphone app to a mental disorder—that is, without providing empirical evaluation data, or lacking a detailed description on the use of the smartphone app for delivering psychological interventions for mental disorders. This includes philosophical papers, vision papers, or papers solely focusing on a technical innovation without an accompanying mobile app and/or targeted mental disorder.

### Classification of Studies

All included studies were classified according to technology- and psychology-related dimensions. Additionally, we recorded the name of the app as well.

The technology-related dimensions included the following: (1) built-in sensors: accelerometer, gyroscope, GPS, microphone, and camera; (2) software features: prompting (any kind of proactive prompting to the patient, for example, reminders, notifications, or motivational messages), health care provider communication (directly communicating with a health care provider through the mobile app), progress (allowing patients to monitor their progress throughout the intervention), assessment (capability to [psychologically] assess the patient, including self-assessment [eg, questionnaire] and automatic assessment [eg, based on smartphone usage patterns]), social (availability of social networking and peer communication, such as forums, chat, messaging, and sharing of experiences or information sources), personalization (ability to customize/personalize some aspects of the mobile app toward the patient), learning (any kind of learning material or support presented to the patient), in situ use (explicit support for using the mobile app in the patient’s natural environment [ecological], that is, which allows real-time [momentary] interventions when they are most needed), gamification (use of game elements and principles), context awareness (capability of detecting the context/environment of the patient, for example, location, ambient sound, and text/call history), virtual reality (VR, use of virtual environments as delivery paradigm), and augmented reality (use of augmented environments as delivery paradigm); and (3) analytics: use of advanced software algorithms in the mobile app or supporting infrastructure (ie, server side)—including machine learning, behavioral analysis, activity analysis, and spatial analysis.

The psychology-related dimensions included the following: (1) mental disorders: the considered mental disorders are based on DSM-5 [33]. In addition to the well-established diagnosis categories from DSM-5, we also considered a *suicidal behavior disorder/non suicidal self-injury* category, as this condition is very well represented in the literature and recognized as a condition for further study in DSM-5 (ie, likely to be included in future versions). In cases where the smartphone app focuses on multiple disorders, we distinguished between (a) comorbid disorders, that is, those specifically focusing on comorbidity, and (b) various disorders, that is, those delivering treatment(s) for different disorders (not co-occurring, that is, in different patients); and (2) approaches to psychotherapy: the different approaches to psychotherapy are based on the existing theories, which guide psychologists through the process of understanding patients and their mental disorders and developing solutions. Taking into account different treatment modalities and psychological frameworks, approaches to psychotherapy fall into eight broad categories: cognitive behavioral therapies, humanistic therapies, systemic therapies, psychoanalysis therapies, third wave therapies, transdiagnostic therapies, positive psychotherapy, and others.

Finally, the study-related dimensions included the assessment type: *Effect*, *Usability/user experience* (short: *Usability/UX*), *Effect and Usability/UX* and *No Assessment*. *Effect* indicates that the authors reported results about the smartphone app’s effects on the participants’ clinical symptomatology. *Usability and user experience*, as defined by ISO 9241-210:2010, that is, the International Standard on Ergonomics of human System Interaction [34], indicates that the authors assessed variables such as usability, user acceptance, opinion and satisfaction, feasibility, and intention to use. *Effect and Usability/UX* denotes that the authors assessed *Effect* as well as *Usability and UX*. Finally, *No Assessment* refers to those cases where no assessment was reported, for example, including study protocols or technical descriptions of the smartphone apps delivering psychological interventions for mental disorders.

### Data and Software Availability

For transparency and reproducibility, we published the resulting data, code, and instructions on GitHub (San Francisco, California) and archived the work in Zenodo [35]. The GitHub repository includes a literate programming document that combines text, data preprocessing, analysis, and visualizations.

## Results

### Study Inclusion

**Figure 1:**
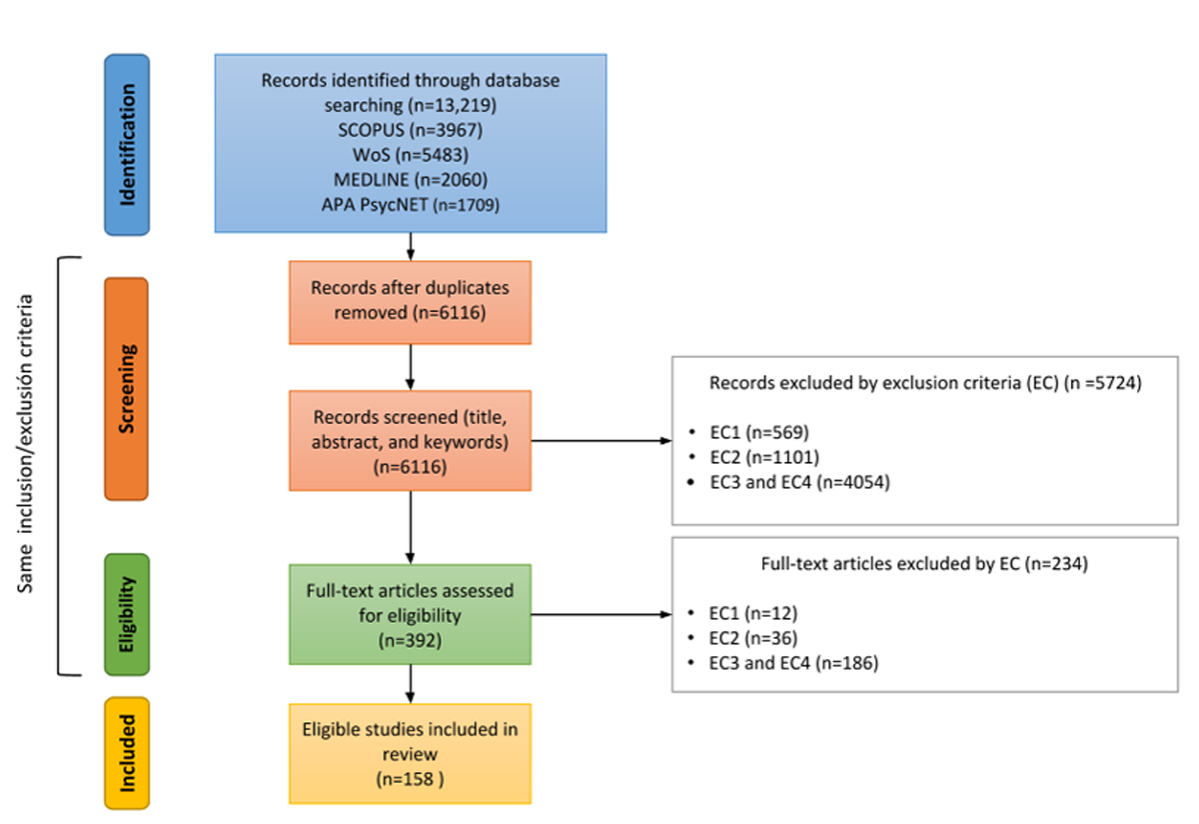
Preferred Reporting Items for Systematic Reviews and Meta-Analyses flow diagram for the systematic review. WoS: Web of Science.

Figure 1 shows the results of the systematic review processes according to the PRISMA data flow chart. During the *identification* phase, we identified 13,219 studies from the four different Web-based sources (Scopus, WoS, APA PsycNET, and MEDLINE), which we reduced to 6116 after removing duplicates. After the *screening* phase, that is, based on title, abstract, and keywords, we retained 392 articles. The *eligibility* assessment, that is, based on the full paper, led to a final set of 158 papers. More details can be found in Figure 1.

### Evolution of Research and Types of Assessment

**Figure 2:**
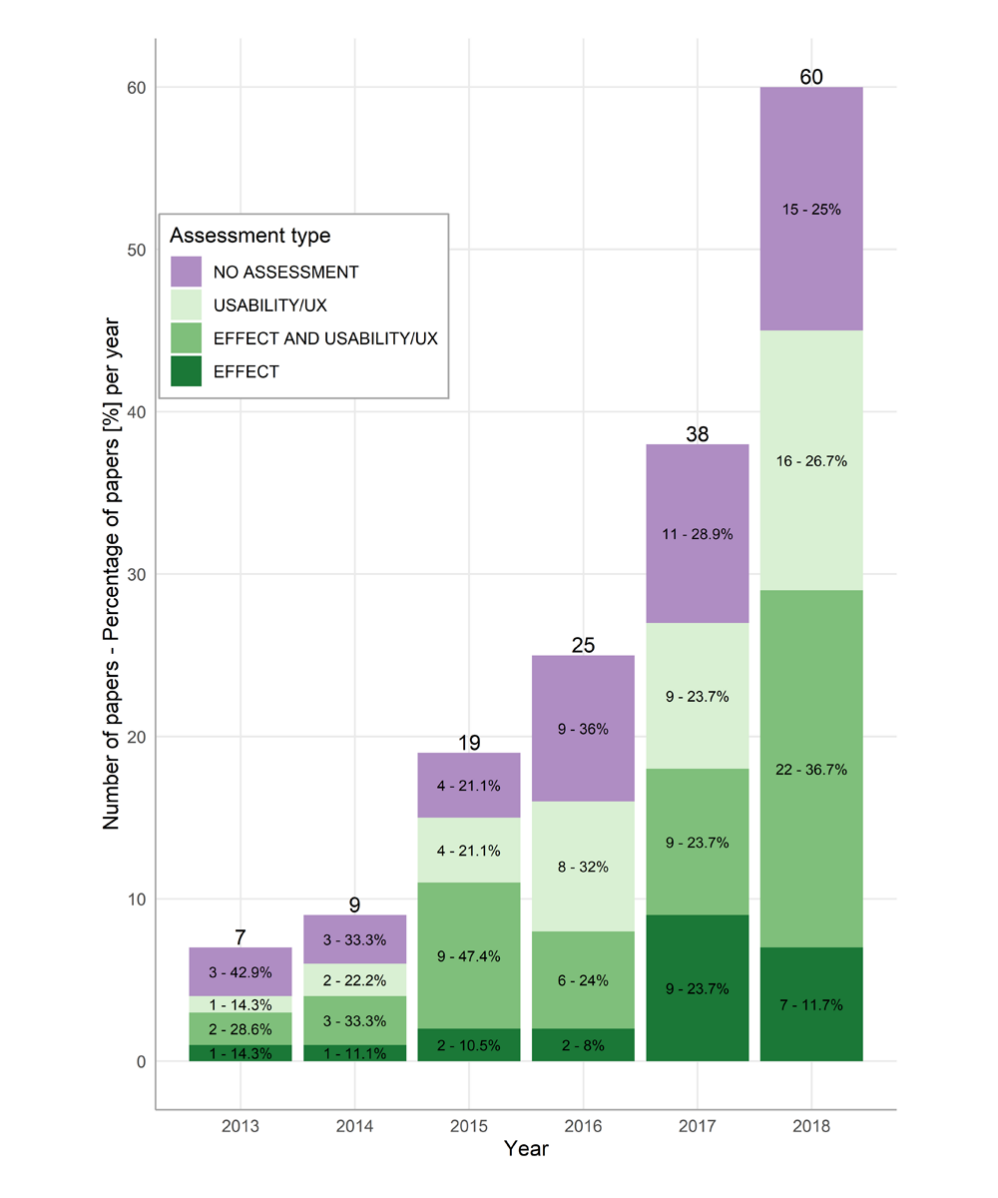
Temporal trend and number of articles published per assessment type.

Figure 2 shows the temporal distribution of research over the study period 2013-2018, along with their reported assessment type. Overall, we observed a positive evolution of the amount of research over time, steadily increasing from only few (7) articles in 2013 to a much larger amount (60) in 2018.

In Table 1, we show the distribution of assessment types for the reviewed studies. The majority of articles (113/158, 71.5%) reported some kind of assessment. Looking at the distribution of assessment types over time (Figure 2; percentages), we observe an overall slow proportional increase of studies with an assessment (2015 appears to have been an outlier). Regarding the type of assessment, we observe that only a fifth of the articles with assessment (22/113, 19.5%) focus specifically on the effect of intervention on clinical symptomatology (15/113, 13.9% of all studies). Although we see an absolute increase over the last 2 years (in line with the overall increase of studies in general), the sharp increase in 2017 could not be confirmed in 2018 in proportional terms. The proportional amount of usability/UX assessments steadily rose over the years, with an outlier in 2016, where it counteracted a sharp drop in mixed assessments. Caution should be taken with interpreting and generalizing these results; additional data over a larger timeframe are needed.

Orthogonal to the general type of assessment, we also considered other characteristics of the assessment—that is, whether it features an RCT over a long timeframe, or a pilot RCT; or supplies less empirically rigorous results, such as qualitative studies, feasibility studies, case studies (eg, n of one clinical trials) or usability studies. From Table 1, we observe that only a small minority of studies performed an RCT assessment (25/113, 22.1% of all studies with assessment; 25/158, 15.8% overall) and only a handful of papers (7/113, 6.2%; 7/158, 4.4% overall) performed a pilot RCT. Moreover, only a minority of all RCTs (9/25, 36%; 9/158, 5.7% overall) and pilot RCTs (2/7, 29%; 2/158, 1.3% overall) were focused specifically on effect assessments.

**Table 1:** Distribution of assessment type.

Assessment types		All, n (%)	RCT^a^, n (%)	Pilot RCT, n (%)
No assessment (total)		45 (28.5)^b^	N/A^c^	N/A
**Assessment (total)**		113 (71.5)^b^	25 (22.1)^d^	7 (6.2)^d^
	Usability/UX^e^	40 (35.4)^d^	2 (8)^f^	0 (0)^f^
	Effect + usability/UX	51 (45.1)^d^	14 (56)^f^	5 (71)^f^
	Effect	22 (19.5)^d^	9 (36)^f^	2 (29)^f^

^a^RCT: randomized controlled trial.

^b^Percentage based on the total number of studies (N=158).

^c^N/A: not applicable.

^d^Percentage based on the number of studies with an assessment (N=113).

^e^UX: user experience.

^f^Percentage based on the number of RCT studies (N=25) and Pilot RCT studies (N=7), respectively.

### Covered Mental Disorders

**Figure 3:**
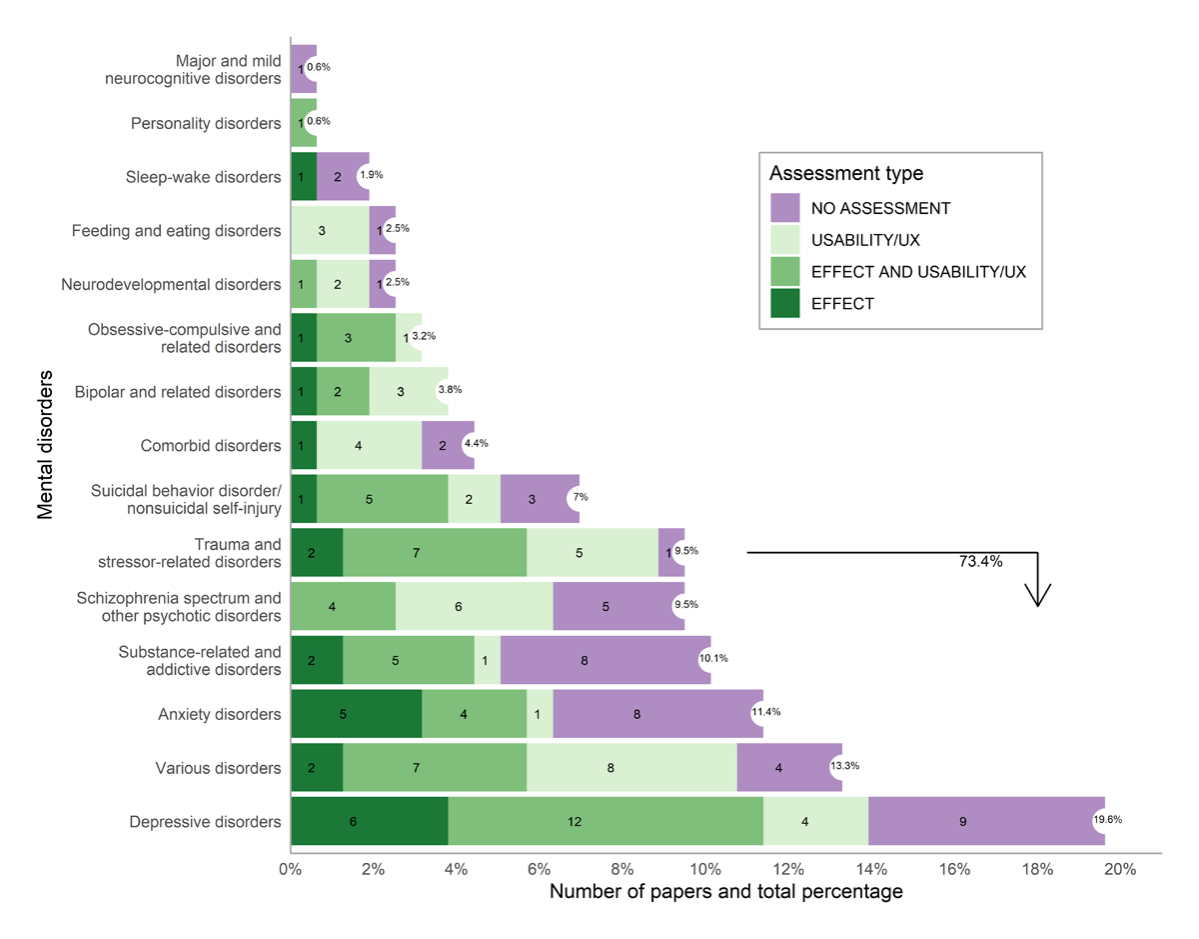
Distribution of articles per mental disorder, categorized according to assessment type. Aggregated results of assessment types: No assessment 45/158 (28.5%), Usability/UX 40/158 (25.3%), Effect + usability/UX 51/158 (32.3%), Effect 22/158 (13.9%).

Figure 3 shows the number of studies per mental disorder, ranked in ascending order and subcategorized according to the type of assessment. *Depressive disorders* (31/158, 19.6%) is the most commonly addressed mental disorder. Note that the category of *various disorders* includes apps addressing multiples disorders, where serious mental illness, *depressive and anxiety disorders* are most represented. Collectively, the top six mental disorders account for 73.4% (116/158) of all studies included in the search. Regarding *comorbid disorders*, we point out that the majority of papers were related to a specific dual pathology, that is, where a psychological disorder coexisted with the abuse of substances. One case in this category was focused on *neurodevelopmental disorders and elimination disorders*. For all remaining mental disorders from DSM-5 [33] (not shown in Figure 3), we did not find studies that met our IC.

**Figure 4:**
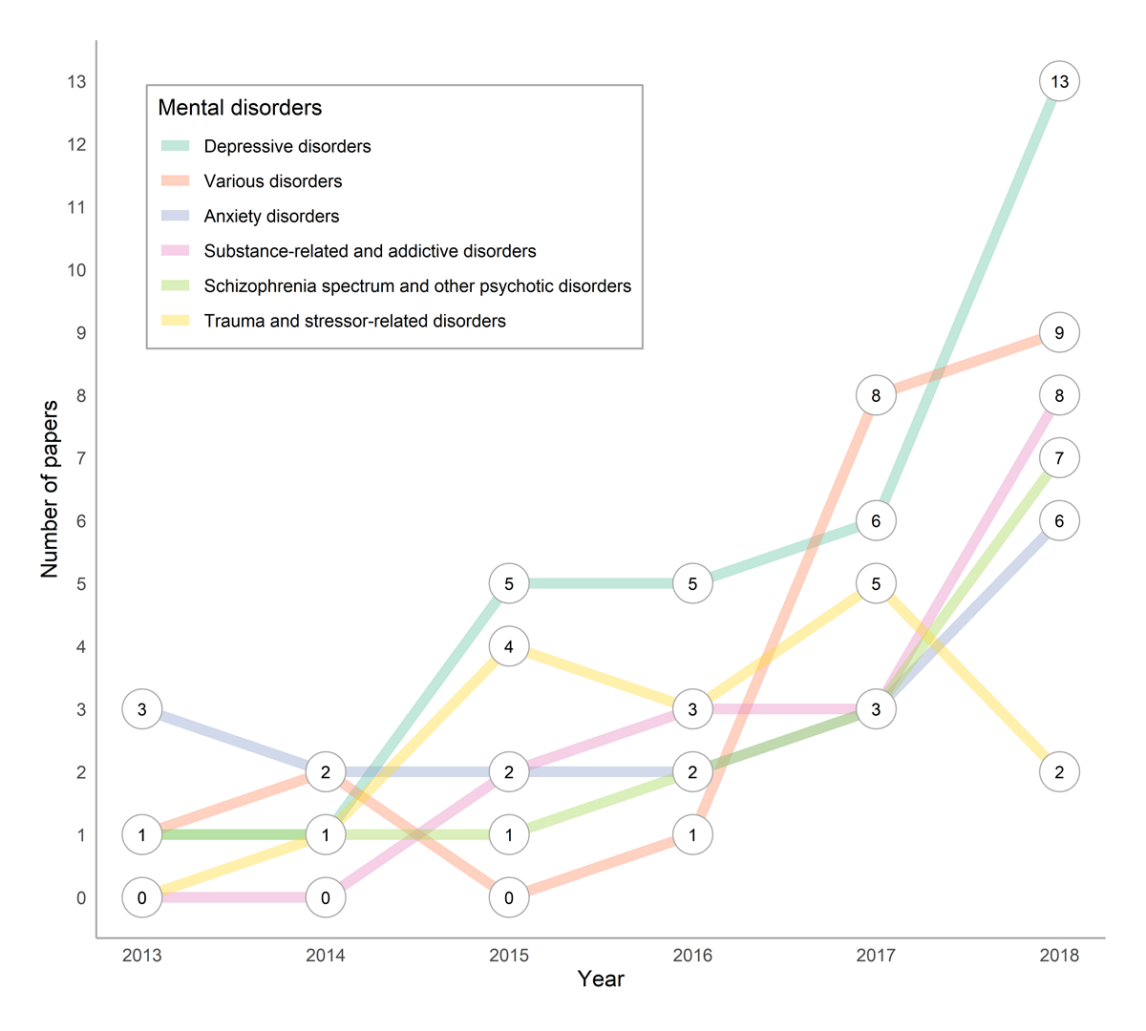
Temporal distribution of articles published for the top 6 mental disorders.

Figure 4 shows the temporal trend of the top six mental disorders targeted by studies over the period 2013-2018. Overall, we observed an increasing number of published articles related to the top six mental disorders over time, with a significant increase since 2015. We also noted that the relative ranking of the top six mental disorders is largely maintained since 2015, with two notable exceptions: *trauma and stressor-related disorders* sharply decreased in 2018, and *various disorders* (ie, the app can be utilized to target multiple independent [noncomorbid] disorders) significantly increased in the last 2 years, reaching the first and second positions, respectively. Finally, we point out the doubling of research on *depressive disorders*—which was already well researched previously—in 2018, and the fact that research on *substance-related and addictive disorders* only started in 2015, yet it has been steadily growing since to reach the third position in 2018.

### Relation Between Assessment Type and Mental Disorder

In Figure 3, we observe multiple effect assessments for all top six disorders, except for *schizophrenia spectrum and other psychotic disorders* (0/15, 0%). The *depressive* and *anxiety disorders* are relatively well studied for effect assessment; 19% (6/31) and 28% (5/18) of assessments pertain to effect, respectively. On the other hand, effect is assessed only marginally for *trauma and stressor-related disorder* (13.3%), *substance-related and addictive disorder* (12%, 2/16), and *various disorders* (9%, 2/21). For less addressed disorders (ie, not in the top six), we only see one or no effect assessment.

Regarding other types of assessment, no clear patterns can be observed, and we fall back to individual observations. Remarkable are the high number of *mixed* assessments for *trauma and stressor-related disorders* (47%, 7/15) and to a lesser extent *depressive disorders* (39%, 12/31); the low number of pure usability/UX assessments for *anxiety disorders* (5%, 1/18), *substance-related and addictive disorders* (6%, 1/16), and *depressive disorders* (13%, 4/31); and the large number of articles without any assessment for *anxiety disorders* (44%, 8/18), which contrasts the high number of effect assessments.

### Coverage of Technical Features Per Mental Disorder

**Figure 5:**
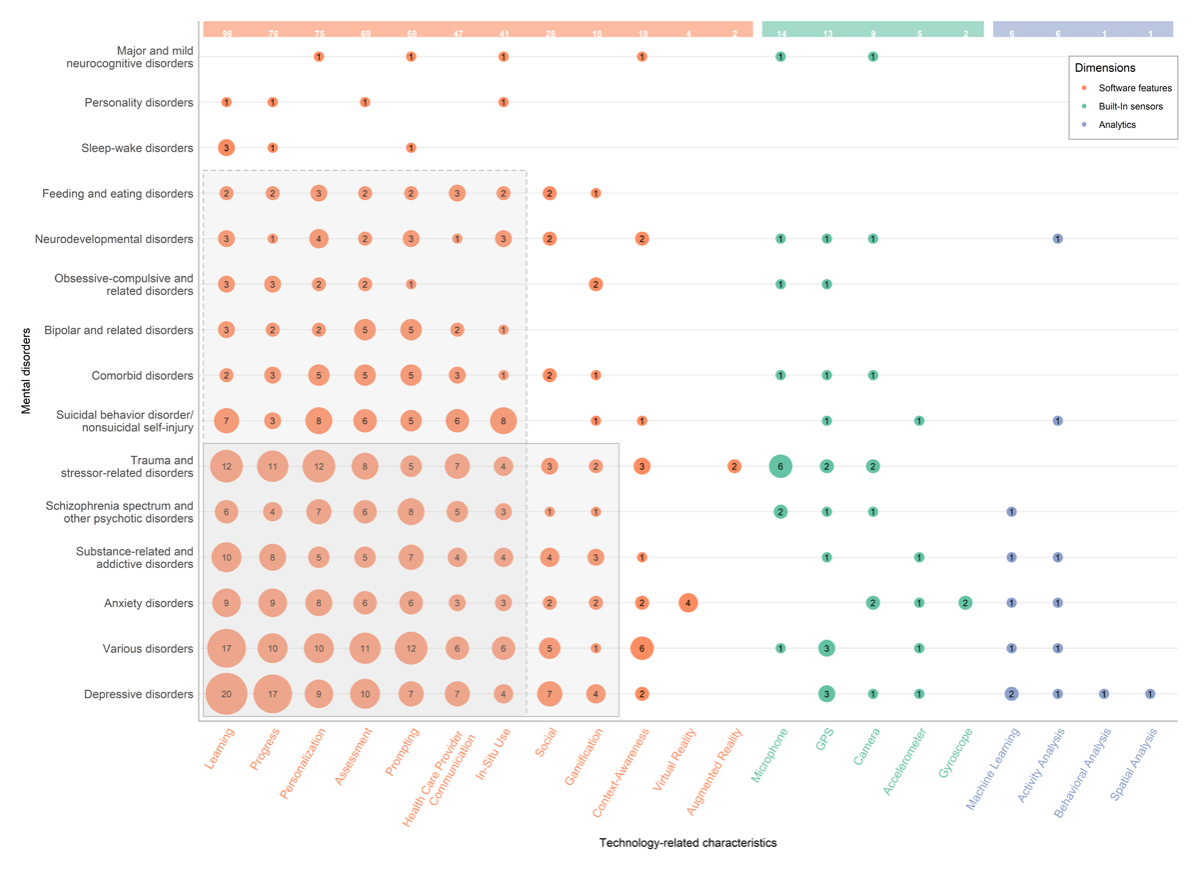
Bubble plot representing technology-related dimensions (software features—orange; built-in sensors—green; analytics—blue) vs mental disorders. Bubble size corresponds with the number of articles.

Figure 5 plots the technology-related dimensions, namely, software features implemented by the studied apps (in orange), the utilized built-in sensors (in green), and analytics (in blue), vs the type of mental disorders. In doing so, the figure shows to which extent, and for which disorder(s), the state of the art is leveraging hardware- and/or software-related smartphone capabilities. Within each technology-related dimension (X axis), features are ranked by their decreasing popularity over all mental disorders (left-right; occurrence count is shown at the top of each column); mental disorders (Y axis) are similarly ordered by decreasing popularity in literature (bottom-up).

We note that larger bubbles tend to be concentrated at the bottom of the graph, as the most popular mental disorders have a higher number of articles, which also tend to cover more varied technical dimensions. The 7×12 vertical rectangle illustrates the top seven software features that are being leveraged for the majority of mental disorders (ie, 12 out of 15). These features are mostly related to intervention-specific features, such as learning and in situ use, and communication features such as prompting. The 9×6 rectangle shows nine software features that have full coverage for the top six mental disorders; it also includes social and gamification features. Regarding the delivery paradigm, virtual and augmented realities are each leveraged for only one mental disorder. Finally, regarding built-in sensors and analytics, we observe a much lower and dispersed coverage without clear patterns (especially for analytics). GPS stands out, with a relatively wide spread over mental disorders (ie, 9 out of 15).

### Concrete Studies Per Mental Disorder and App Name

For the benefit of the research community, Table 2 lists the concrete studies (by app name, when available; if the app name was not found, we put N/A) per mental disorder. Apps that are the subject of multiple studies are shown in *italics*. On the one hand, it can be observed that, independent of the mental disorder, most studies utilized a custom-made app, which was not being assessed in other studies. On the other hand, this implies that there exists a wide variety of apps, even for the same mental disorder. Remarkably, for *trauma and stressor-related disorders*, most apps were assessed in multiple studies. Highlighting some interesting cases, Koroko-App (*depressive disorders*) is an app that was rigorously tested both for effect and usability/UX; a study protocol was published, followed by two RCTs assessing effect and usability/UX issues. Other apps, such as post-traumatic stress disorder coach and Simply Yoga (*trauma and stressor-related disorders*), combine RCTs with other type(s) of assessments for effect and usability/UX. Some apps such as the Blue Ice app (*suicidal behavior disorders/nonsuicidal self-injury*) published assessments using non-RCT designs.

**Table 2:** Apps and studies grouped by mental disorder (apps covered in multiple publications are in italics).

Mental disorder	References by app
Major and mild neurocognitive disorders	Rico [36]
Personality disorders	EMOTEO [37]
Sleep-wake disorders	Sleepcare [38], SleepIO [39,40]
Feeding and eating disorders	Jorvie [41], Student Bodies–Eating Disorders [42], *Recovery Record* [43,44]
Neurodevelopmental disorders	iCanLearn [45], LifePal [46], My MFG [47], TimeOut [48]
Obsessive-compulsive and related disorders	Geo-Feedback App [49], GGOC [50], Live OCD Free [51], Mayo Clinic Anxiety Coach [52], RAW HAND [53]
Bipolar and related disorders	MyT [54], PRISM [55], *SIMPLe* [56-59]
Comorbid disorders	CASA-CHESS [60], Enuresis Trainer [61], Learn To Quit [62], SMI-CM [63], Stay Quit Coach [64], Stop-Cannabis [65], N/A^a^ [66]
Suicidal behavior disorder/nonsuicidal self-injury	BackUp; mEMA [67], BeyondNow [68], BRITE [69], iBobbly [70], *Virtual Hope Box (VHB)* [71,72], N/A [73,74], *Blue Ice* [75-77]
Trauma and stressor-related disorders	RELAX [78], N/A [79], *Life Armor; PE Coach; Positive Activity Jackpot; Eventful; Tactical Breather; VHB; Daily Yoga; Simply Yoga* [79,80]*,* *PE Coach* [81-84], *PTSD Coach* [85-91]
Schizophrenia spectrum and other psychotic disorders	iCOPE [92], MindFrame [93], movisenseXS [94], RealLife Exp [95], SlowMo [96], TechCare [97], Temstem [98], *Actissist* [99,100], *FOCUS* [101,102], *Heal Your Mind* [103], *PRIME* [104,105]
Substance-related and addictive disorders	Drink Less [106], Fit&Sobber [107], Mind the Moment [108], S-Health [109], SEVA [110], SmartQuit [111], Smoke Mind [112], Social-Local-Mobile [113], *A-CHESS* [114,115], *CET App* [116,117], *Kick.it* [118,119], *Smart-T* [120,121]
Anxiety disorders	Agoraphobia Free; Stress Free [122], Ångesthjälpen [123], AnxietyCoach [124], CBT Assistant [125], Challenger [126], Lantern [127], PsychAssist [128], Public Speech Trainer (PST) [129], SmartCAT [130], *GET.ON PAPP* [131,132], N/A [133-139]
Various disorders	ACT Daily APP [140], FOCUS [141], Headspace [142], iBobbly [143], iCare-stress [144], IntelliCare Suite [145], MoodMission [146], MoodTrainer [147], myCompass [148], PeerTECH App [149], Pocket Skills [150], Sinasprite [151], SmartCAT [152], SPIRIT [153], The Moment [154], TODAY! [155], Wellframe [156], WellWave [157], N/A [149], *WorkingWell* [158,159]
Depressive disorders	7Cups [160], Be Good to Yourself [18], BlueWatch [161], Dcombat [162], Get Happy Program [163], HeadGear [164], iCare Prevent [165], MedLink [166], Mobile Sensing and Support [167], MoodHacker [168], Moodivate [169], MyGamePlan [170], PRIME-D [171], Push-D [172], SocioEmpathy [173], SPSRS [174], SuperBetter [175], The Sound Advice [176], Thought Challenger [177], TODAC [178], *Kokoro-App* [179-181], N/A [19,80,182-187]

^a^N/A: app name not available/not mentioned.

### Overview of Randomized Controlled Trial Assessments

In Multimedia Appendix 3, we provide a comprehensive table with key variables of (pilot) RCT studies, including their main characteristics and results. We found a total of 32 rigorous assessment designs, with 7 pilot RCTs (22%) and 25 RCTs (78%). Below, we discuss the psychotherapy approaches (ie, treatment modalities) found in these assessments, temporal evolution of assessment types in RCTs, and notable observations on covered mental disorders.

Regarding approaches to psychotherapy, we found that 80.4% (127/158) of all included studies follow a cognitive behavioral therapy (CBT) psychological framework; 21.3% (27/127) of these additionally include third wave therapy techniques such as mindfulness or acceptance, or commitment therapy techniques. There are 13 apps fully based on a third wave therapy. Two apps are based on behavioral activation and physical activity. For the remaining mobile apps, no information regarding their psychological framework was reported in the article. When specifically considering the pilot RCT and the RCT studies, we observed the same trend: the majority is based on the CBT psychological framework, followed by studies which combine CBT with third wave therapy techniques. When looking at the overall temporal evolution of RCT studies, we noticed an even spread of combined effect and usability/UX evaluations over time. For effect studies, however, we observed that the majority took place in the last 2 years.

The most commonly addressed disorders by RCT studies were *depressive disorders* (9/25, 36% of the RCT studies). The majority of these studies showed that participants who received intervention apps significantly improved their symptoms (depression, anxiety, etc) compared with the waiting list [18,175], alternative care [168], or control conditions [178,180]. Furthermore, studies with follow-ups showed that the treatment benefits were maintained [168,178]. One of the RCTs compared an intervention app with treatment as usual (ie, therapist); the results showed that, at posttreatment, the clinical variables did not differ between groups [186]. Another study compared two different apps, each featuring a different approach to psychotherapy (behavioral activation and mindfulness); the results showed that both apps were useful and did not differ significantly from one another [157]. The second most addressed disorder involved *anxiety disorders* (4/25, 16% of the RCT studies). Here, the results showed the same trend as for depressive disorders; participants who received intervention apps improved their symptoms significantly compared with the waiting list [123,126,139]. Furthermore, in studies with follow-ups, treatment benefits were maintained [126,139]. One of the studies compared two different intervention apps with different intervention targets, that is, agoraphobia vs general anxiety symptoms, for managing agoraphobia [122]. The results showed reductions in symptom severity over time that was statistically significant without differences between both apps.

For *schizophrenia spectrum and other psychotic disorders* (3/25, 12% of the RCT studies), we found that 2 RCTs used the same app [104,105]. Results showed significant improvements in clinical symptoms posttrial compared with the waiting list, and also good acceptability [104,105]. A third app also showed positive results at posttreatment [100]. For *substance-related and addictive disorders*, we found one RCT; the study found improvements in alcohol outcomes for the participants [106]. Several other disorders were also only covered by one RCT. In case of *sleep-wake disorders*, the intervention app produced significant improvements in insomnia severity and sleep efficiency compared with the waiting list [38]. For *suicidal behavior disorder/nonsuicidal self-injury disorders*, the intervention app reported a significantly improved ability to cope with unpleasant emotions and thoughts compared with the control group [71]. In case of *bipolar and related disorders*, participants in the intervention app group showed significantly greater reductions in depressive symptoms [55]. For *trauma and stressor-related disorders*, RCT studies showed slightly less promising results. Usage of an intervention app did not result in significantly better outcomes compared with other active control conditions [79]; still, outcomes were better when compared with the waiting list condition [85].

## Discussion

### Principal Findings

In general, we infer a growing interest in utilizing smartphone apps for delivering psychological treatments, with research increasing from only a few (7) articles in 2013 to an order of magnitude more (60) in 2018. This is a promising trend, as these apps can complement therapist-led psychological treatments and, hence, increase their efficacy and availability. When delegating (part of) psychological treatment to smartphone-based interventions, the need for face-to-face sessions and manual follow-up is decreased, which, in turn, lowers costs and reduces waiting lists in the public health system. According to the mental health workforce breakdown (by the World Health Organization region), there are only 4.6 psychologists per 100,000 inhabitants in Europe [188]. For Spain, studies have reported wait times of more than 45 days before the first psychological assistance by a clinical psychologist or psychiatrist [188], and a frequency of face-to-face sessions of around once a month [189]. Moreover, leveraging smartphones’ capabilities enables ecological momentary interventions (EMI), whereby patients are able to access psychological care when and where they need it most, in their natural environment and daily routines [190-192]. Below, we discuss our concrete observations on the assessment types of included studies, coverage of mental disorders, and technical features.

#### Evolution of Research and Types of Assessment

It is a promising sign that overall, the number of articles with some sort of assessment is slowly increasing. Furthermore, we observe that proportionally, there is a much higher number of studies with an evaluation of only usability/UX, compared with only effect. Usability factors have been widely recognized as key factors to enhance the acceptance of information and communication technologies (ICT) tools; on the basis of the technology acceptance model, authors have suggested that the intention to use a product in the future is strongly correlated with its ease of use [193,194]. Hence, initial efforts to research and ensure the usability of new ICT tools are essential. At the same time, we observe an overall much lower number (less than half compared with usability/UX) of studies that explicitly assesses the effect of smartphone interventions on clinical symptomatology (despite a peak in 2017). Yet, it is specifically this type of studies, focusing on the (long-term) clinical effects of the intervention, that are needed to demonstrate efficacy, and increase therapists’ and patients’ trust in smartphone-based interventions.

Moreover, RCTs, which are considered the gold standard of experiment design in mental health (and medicine in general), are only minimally represented in the literature (22/158, 15.8% of articles overall). Among them, we see an even spread of combined effect and usability/UX evaluations over time; for studies specifically focusing on effect, however, the majority took place in the last 2 years (with a peak in 2017). This is a promising sign, although there are still relatively few effect studies (see Multimedia Appendix 3). Furthermore, the most commonly addressed disorders using the RCT methodology are depressive disorders, followed by anxiety disorders. It is, thus, important to carry out more RCTs to prove mental health apps’ efficacy in treating other mental disorders, and to study the satisfaction and experience of the patients using these apps. Moreover, to draw rigorous and trustworthy conclusions on the clinical efficacy of smartphone apps, more long-term RCT studies will be needed (eg, to better measure the effects of attrition). Similarly, we observe a distinct lack of cross-validation studies, with only a few apps having been studied in multiple articles (19/138, 13.8%). One could note that this phenomenon is correlated with the lack of rigorous long-term studies on smartphone interventions—a single, multiyear study would warrant multiple articles for a single smartphone intervention on study protocol, usability evaluation, and effect studies at multiple intervals. Particularly when utilizing novel technological features, rigorous assessment studies are needed to validate their potential for psychological interventions and encourage further research in the field. A stronger cooperation between research groups could increase the resources needed for such long-term psychological intervention studies.

#### Covered Mental Disorders

To an extent, the coverage of mental disorders in the relevant literature seems to be in line with their real-world prevalence. This holds, in particular, for depressive and anxiety disorders, commonly called emotional disorders [195]; they (1) represent the first and third most covered disorders in the literature (we point out that *various disorders* include apps addressing multiples disorders, where depressive and anxiety disorders, in addition to serious mental illness, are most represented), with the research on *depressive disorders* being doubled in 2018; and (2) they are known to affect the most people worldwide. For mental disorders with highest prevalence among people [196,197], lifetime prevalence has been estimated at 28.8% for anxiety disorders, 20.8% for mood disorders (including 16.6% for depressive disorders, which are a mood disorder), 24.8% for impulse-control disorders, and 14.6% for substance use disorders. Estimated 12-month prevalence follows a similar trend: anxiety disorders are the most prevalent class with 18.1%, followed by mood disorders with 9.5% (including 6.7% for depressive disorders), impulse-control disorders (8.9%), and substance disorders (3.8%) [196,197]. Hence, according to the psychological literature [198,199], the three most prevalent mental disorders include anxiety, mood (including depressive disorders), and substance disorders. Indeed, these similarly make up our top four of most covered mental disorders in smartphone intervention studies. Depressive and anxiety disorders reduce a patient’s psychosocial functioning and quality of life [198,200], and are associated with important personal, social, and economic repercussions [199,201]. Other ICT technologies for delivering psychological treatments, such as internet and Web-based programs, are also mostly focused on depressive and anxiety disorders [202,203]; this might also have had an influence on the proliferation of smartphone-based interventions. Although determining the underlying factor(s) behind the distribution of addressed mental disorders in the literature is certainly an interesting exercise, we consider this beyond the scope of this paper.

Beyond depressive and anxiety disorders, the literature is heavily focused on only a small number of disorders; six mental disorders account for approximately 73.4% (116/158) of research. On the other hand, more than half of the categories of mental disorders listed in DSM-5 (15) are fully excluded or very scarcely represented (<3%). Clearly, there is an opportunity, as well as an acute need, to pay more attention to the whole breadth of mental disorders—that is, including those that are less prevalent—to help as many people as possible. Some of these less prevalent disorders, such as personality disorders, often have a higher severity that may lead to extreme consequences. For instance, borderline personality disorder affects only 2% to 6% of the population [204,205], but its mortality rate by suicide is one of the highest in the world among people with psychiatric disorders [206].

#### Coverage of Technical Dimensions

When looking at technical dimensions, more traditional software features (see 7×12 vertical rectangle in Figure 5) are much more utilized than the novel sensing or analytical capabilities of smartphones. One may argue that these top seven features, which involve intervention-specific features (eg, learning) and communication features (eg, prompting), do not offer a significant advancement over the prior state of the art. Indeed, many previous studies that leveraged (nonsmartphone) mobile phones supported learning by displaying psychoeducational content [56,58,140], receiving tips/reminders via SMS [47,149], using (bidirectional) SMS communication to perform (in situ) assessment [149], or telephone calls to health care providers [75,81,145]. Notwithstanding these observations, even this rather conservative transition to smartphones has enabled interventions that are out of reach for classic mobile phones. Research leveraging smartphones have exploited larger screen resolutions and multimedia capabilities to provide multimodal learning materials, using audio and video guides [122,184], pictures [71,75-77,87,154], audio [71,76,87], music [75-77], and video [71,77,174]. Some authors have leveraged the improved connectivity and ubiquity of smartphones to offer access to entire Web-based libraries of learning materials [152,184]; others utilize in-app prompting as intervention techniques, for example, sending reminders to use the app [76,85,116,178], motivational messages [47], or messages from the therapist [80,103]. We found studies that exploit the improved interactivity of smartphones to provide interactive quizzes for training skills and improved learning [62,152], assessments for panic attacks [133], suicidal intentions [143], symptoms of various disorders [54,120,121], and communication with therapists [37,58,92,133] or other users [79,104] through message/chat. Furthermore, aside from being better supported by smartphone capabilities, many of these psychological smartphone interventions are available at the touch of a button, instead of relying on receipt of SMS or phone calls.

That said, most studies still only scratch the surface of advanced smartphone capabilities. This is particularly apparent in the relatively low coverage of context awareness, that is, leveraging sensors to detect and react upon the current state of patients and their environments. We argue that such context awareness is a key ingredient of true EMI. Indeed, although EMI are meant to proactively issue suitable therapeutic interventions at the right time and place, most EMI studies consider smartphones merely as a tool for manually accessing interventions, or receiving predefined interventions at set time intervals, at any moment and place. We found a very limited number of smartphone-based studies leveraging external sensors for recording physiological parameters: measuring heart rate for detecting physiological arousal in the context of anger management [78], for instance. Similarly, we found very low coverage of analytics-based studies that could support advanced context awareness, for example, learning and assessing mental states based on physiological, environment, activity, and/or behavioral contexts. In our opinion, studies that progressively use internal and/or external smartphone sensors, possibly combined with advanced analytics, are a useful step toward realizing the full potential of EMI, where relevant events are detected through analysis of sensor readings, and acted upon by triggering suitable, personalized interventions when they are needed. The general hesitance toward context-aware EMI could be explained by the lack of validated computerized psychological models for assessing mental states based on patient context (including physiological, environment, and activity factors), as well as the need to combine technologically advanced solutions (ie, use of sensors, context awareness, and analytics).

Through their improved modalities (screen and sound), interactivity, and computational resources, smartphones enable novel intervention delivery paradigms, including virtual and augmented realities. However, these have found very limited coverage in the literature. We found a few individual studies, for example, utilizing a mobile VR system to help patients coping with agoraphobia by guiding an avatar through real-life simulations in a game-based setting [122]. Beyond exposure-type therapies, Repetto et al [137] utilized VR techniques to cope with generalized anxiety disorders, leveraging biofeedback to regulate features of the virtual world (eg, current heart rate). As mentioned before, the seeming lack of nontraditional intervention methods may be because of the lack of validated psychological models for supplying evidence-based VR or augmented reality, and/or the technical difficulty of novel delivery paradigms.

Given the lack of studies on these topics, we believe that there lies a huge potential for future research in utilizing technologically advanced solutions (ie, sensors, context awareness, and alternative delivery paradigms) to deliver smartphone-based psychological interventions tailored to the patient’s current health context.

#### Barriers to Implementation and Patient Risks

Despite our advocation for further research in the utilization of smartphone features to advance treatments for mental disorders, we note that technological innovation should not constitute a goal in itself. It must provide a distinct advantage toward patient care, such as improved mental health care access, that is, a broader reach and lower barriers; increased assessment frequency and accuracy; lower cost; improved efficacy; and immediate access to care, when and where the patient needs it most. These intended benefits must be balanced with possible adverse effects, and the use of treatment modalities, including advanced technical features, needs to be carefully contemplated.

Here, we point out possible risks and barriers to implementation of smartphone-based interventions. The lack of research evidence on the *effectiveness of mobile health apps* is likely the most important issue to consider [207,208]. As shown in Table 1, only a limited number of smartphone apps are validated using an RCT, and we observed a distinct lack of cross-validation studies, with only a few apps having been assessed in multiple studies. We further point out that this analysis only covers apps presented in the literature and not the many thousands of other, nonvalidated apps in popular app stores. There is a clear risk in using nonvalidated apps, as there is no evidence that they have any therapeutic effect, and they may even worsen the patient’s condition. For example, Baron et al [209] noted that low-accuracy sleep trackers to self-diagnose sleep disturbances caused patients to be overly concerned on getting the perfect sleep (*orthosomnia*), which may have exacerbated their insomnia.

The ability to protect the *privacy and confidentiality* of patient information is pivotal as well [207,210-212] and may form a barrier to adoption. Many health-related apps collect a large amount of demographic, medical, and lifestyle information, as well as data on users’ daily routines and practices [213]. Some apps have even been found to collect user data unrelated to the app’s purpose [211]. Despite the importance of privacy with respect to (mental) health data, several researchers have found that a significant portion (31%-49%) of studied mental health apps does not include a privacy policy [210,214]. Collected information may be distributed to (third-party) services for storage and analysis. Moreover, unregulated apps may even pass on this information to unidentified parties, for example, for advertising purposes [210,211]. In a study of depression and smoking cessation mobile apps, it was observed that the majority (81%) of the studied apps transmitted data for advertising and marketing purposes, but only 59% of the apps disclosed this in their privacy policy [210]. In all the aforementioned cases, there is a risk that personal data are being transmitted over insecure network connections [215,216]. Furthermore, there is the possibility of loss, theft, or malfunction of the mobile device [5], or cybercriminals specifically targeting health information [207], all of which could result in the loss of sensitive data.

Toward addressing these two issues, some authors suggest that mental health professionals should screen the apps they recommend for privacy issues [217]; however, this does not seem like a feasible or robust solution. Olff [218] proposed a disclaimer for apps that have not been validated. Taking it one step further, certification processes for mobile (mental) health apps have recently been instigated in the European Union (EU) and the United States, even though they do not apply to all apps. Under the 2017 EU Medical Device Regulation [219], mobile apps with a medical intended purpose require a CE marking. Devices (apps) may be differently classified depending on the purpose and risk they pose, yielding more or less stringent regulations regarding quality, control, or development process. The EU General Data Protection Regulation [220] has furthermore motivated a number of app developers to improve transparency on privacy policy, although confusion remains on its applicability outside the EU [211]. Similarly, in the United States, the Food and Drug Administration policy for mobile medical apps [221] stipulates that any software that is utilized for the diagnosis of disease or other conditions, or the cure, mitigation, treatment, or prevention of disease, constitutes a medical device, regardless of the platform on which it is run (eg, desktop or mobile). The applicable regulations depend on the particular functionality. Given the recency of these (updated) regulations, their rate of adoption and effects on efficacy, safety, privacy, and usability of mHealth apps remain to be seen, and we expect the regulations to be further clarified, refined, and extended in scope in the future.

Further barriers to implementation include a lack of sufficient general, digital, and health literacy levels [207] and a digital divide [222], the view and attitude of practitioners and patients toward the use of mobile mental health interventions [223,224], availability and awareness of evidence-based apps [225], economic and other associated costs [226], and user acceptance and usability [226]. For further elaboration on barriers and facilitators to implementation of mental health care apps, we refer the readers to Lipschitz et al [225] and Simblett et al [226].

#### Recommendations for the Research Community

In line with our findings, we propose four recommendations for the research community to further develop and advance the field of smartphone-based psychological interventions:

- Attention for less covered disorders: The majority of research (approximately 73%) pertains to the top six covered mental disorders, four of which coincide with highly prevalent mental disorders. On the other hand, more than half of the DSM-5 recognized mental disorders are not or very scarcely covered. We call upon the research community to invest into covering the full breadth of mental disorders.
- Attention for advanced technical and software-based solutions: Many smartphone-based psychological interventions merely translate traditional and electronic health (eHealth) solutions to smartphones; that is, these interventions do not fully exploit their capabilities. Concretely, the use of sensors and corresponding context awareness, particularly to promote EMI, the exploration of alternative delivery paradigms such as virtual or augmented reality, and more advanced analytical methods are scarcely investigated. We call upon the research community to explore beyond traditional strategies, toward leveraging advanced technological features to improve mHealth interventions.
- Multidisciplinary approaches: To fully exploit the smartphone’s capability as a pervasive, ubiquitously connected, sensor-packed computing platform to deliver innovative, real-time, and in situ psychological interventions, both the domain knowledge of psychologists and the technical expertise of computer scientists are needed. Hence, we call for multidisciplinary collaborations as to not let technical difficulties, or lack of psychological knowledge on mental disorders, hinder advances and novelties in the field.
- Validation toward effect: Although we uncovered, at least in absolute numbers, a slight increase in effect validations and RCT-based effect assessments during the last 2 years combined, they are still underrepresented (particularly RCTs). Hence, there exists a need to rigorously validate smartphone-based psychological treatments for effect. Especially when utilizing advanced technical features (eg, context awareness, analytics, and alternative delivery paradigms), effect validation may increase trust and spark further research in such novel types of interventions. We call upon the research community to augment efforts in rigorous effect assessment, to allow transfer of research into practice.

Despite our call for research in technical innovation and its broader applicability and validation, we note that the eventual use of such advanced technical features—or any technological aid in psychological interventions for that matter—needs to be carefully balanced with the characteristics and needs of the individual patient.

### Strengths and Limitations

The main strength and novelty of this study is that it explored and summarized, considering a wide range of technical characteristics, the current state of the art in smartphone-based interventions for mental disorders. We hereby provide a broad overview of the field (1) covering the full spectrum of mental disorders as classified in the latest version of DSM, rather than focusing on a specific mental illness as done in previous studies; and (2) exposing technical features used to realize smartphone-based treatments. Consequently, this contribution is highly innovative as a synergetic study targeting mental health research and recent developments in mobile sensing and computing. Further strengths of this study include the use of four different bibliographic sources for a comprehensive coverage of the research and literature, and the methodological process based on pairs of multidisciplinary researchers for the selection, validation, and classification of the literature.

As any systematic study, search term specification may lack other relevant terms not considered by the authors, and searches only covered the literature published in English. Therefore, there always exists a risk to not fully identify all relevant studies. Classification of studies may also be prone to error. To reduce this risk, we used pairs of researchers from different disciplines with a requirement of interrater agreement.

### Conclusions

We presented a comprehensive systematic review of the state of the art in smartphone-based psychological interventions, with a synergetic focus on psychology-related issues, such as mental disorders and type of assessment, as well as technological features, such as software features and device sensors. Our results show a rapid increase over recent years in the number of psychological interventions for various mental disorders using smartphone-based apps. It captures how depressive and anxiety disorders are primarily covered, in line with their real-world prevalence. The top six of mental disorders together account for approximately three-quarters of coverage in the literature, while over half are not or very scarcely covered. This implies the need for further research on smartphone interventions for the full breadth of mental disorders to help as many affected people as possible. On the technical side, the review highlights a group of software features related to intervention (eg, learning and in situ use) and communication (eg, prompting) deployed in smartphone interventions that mostly mimic more traditional mobile phone and eHealth solutions. More innovative use of smartphones’ capabilities, such as sensing, alternative delivery paradigms, and advanced analytics, are only scarcely present in the literature, despite their potential for advancing solutions such as EMI. With regard to studies including an assessment, we found that there is an overall slow proportional increase, with significantly more usability/UX compared with effect studies. RCT studies are still a small minority. They mostly deal with depressive and anxiety disorders. Over the last 2 years, there are promising yet inconclusive signs of more effect studies.

## Appendix

Multimedia Appendix 1 Keywords.

Multimedia Appendix 2 Search queries.

Multimedia Appendix 3 Key variables for randomized controlled trials or pilot randomized controlled trial studies.

## Abbreviations

APA: American Psychological Association
CBT: cognitive behavioral therapy
DSM: Diagnostic and Statistical Manual of Mental Disorders
EC: exclusion criteria
eHealth: electronic health
EMI: ecological momentary intervention
EU: European Union
IC: inclusion criteria
ICT: information and communication technologies
MEDLINE: Medical Literature Analysis and Retrieval System Online
mHealth: mobile health
PRISMA: Preferred Reporting Items for Systematic Reviews and Meta-Analyses
RCT: randomized controlled trial
UX: user experience
VR: virtual reality
WoS: Web of Science
