# Supplementary material for: Smartphone Apps for the Treatment of Mental Disorders: Systematic Review
Source: JMIR Mhealth Uhealth. 2020 Apr 2;8(4):e14897. doi: 10.2196/14897 (PMC7163422; doi:10.2196/14897)
Supplement: Multimedia Appendix 1 [file mhealth_v8i4e14897_app1.docx]

## Supplementary Material A: Keywords

## The list of keywords related to psychology were:

- mental (and also e-mental)
- psycholog* (i.e. psychology, psychological)
- psychiatric
- emotional
- health (and also e-health, u-health)
- treatment(s) (and also pretreatment, post-treatment)
- disorder(s)
- intervention(s)
- therapy(ies)
- distress,
- affection
- depressi* (i.e. depression, depressive)
- anxiety,
- ecological momentary intervention

## The list of keywords related to computer science were:

- cell (and cell-phone, cellphone)
- mobile
- smart (and smartphone)
- portable
- phone(s)
- device(s)
- app(s)
- applicat* (i.e. application)
- mhealth
- uhealth
- ehealth
- emental
- android
- iphone
