# Supplementary material for: Smartphone Apps for the Treatment of Mental Disorders: Systematic Review
Source: JMIR Mhealth Uhealth. 2020 Apr 2;8(4):e14897. doi: 10.2196/14897 (PMC7163422; doi:10.2196/14897)
Supplement: Multimedia Appendix 2 [file mhealth_v8i4e14897_app2.docx]

## Supplementary Material B: Search Queries

Queries used boolean operators (AND, OR), proximity operators (NEAR/3, W/3) and wildcard characters supported by each citation database/information source. All searches were against title, keywords and abstract fields, and time frame was between 2013-2018. The queries were launched on March 9^th^ 2018, covering results from 2013 until March 2018; and re-launched on July 13^th^ 2019 to cover the full year of 2018.

### SCOPUS search

We ran one search against SCOPUS as below.

( TITLE-ABS-KEY ( ( ( cell OR mobile OR smartphone* OR smart OR portable ) W/3 ( phone* OR device* OR app OR apps OR applicat* ) ) OR mhealth OR uhealth OR ehealth OR emental OR android OR iphone OR mobile OR app OR apps) AND DOCTYPE ( ar OR cp ) AND PUBYEAR > 2012 ) AND ( TITLE-ABS-KEY ( ( ( mental OR psycholog* OR psychiatric OR emotional ) W/3 ( health OR treatment* OR disorder* OR intervention* OR therapy OR distress OR affection ) ) OR depressi* OR anxiety OR "Ecological Momentary Intervention" ) AND DOCTYPE ( ar OR cp ) AND PUBYEAR > 2012 AND LANGUAGE ( english ) )

### WoS searches

We run a search with the computer science related keywords (WoS Search #1) and a second one with the psychology related keywords (WoS Search #2). The third search (WoS Search #3) performed the intersection of the previous two. All searches used the following WoS collections /indices: CORE collection, Science Citation Index Expanded (SCI-EXPANDED) --1900-presente, Social Sciences Citation Index (SSCI) --1956-present, Arts & Humanities Citation Index (A&HCI) --1975-present, Conference Proceedings Citation Index- Science (CPCI-S) --1990-present, Conference Proceedings Citation Index- Social Science & Humanities (CPCI-SSH) --1990-present, Emerging Sources Citation Index (ESCI) --2015-present.

#### WoS Search #1:

(((TS=(( ( cell OR mobile OR smartphone* OR smart OR portable ) NEAR/3 ( phone* OR device* OR app OR apps OR applicat* ) ) OR mhealth OR uhealth OR ehealth OR emental OR android OR iphone OR mobile OR app OR apps )))) AND Language: (English) AND Document Types: (Article OR Proceedings Paper)

#### WoS Search #2:

(((TS=((( mental OR psycholog* OR psychiatric OR emotional ) NEAR/3 ( health OR treatment* OR disorder* OR intervention* OR therapy OR distress OR affection ) ) OR depressi* OR anxiety OR "Ecological Momentary Intervention" )))) AND Language: (English) AND Document Types: (Article OR Proceedings Paper)

#### WoS Search #3:

(#1 AND #2) AND Language: (English) AND Document Type: (Article OR Proceedings Paper)

### MEDLINE searches

Like in WoS searchers, we conducted three separated queries to get the record form MEDLINE that satisfied both set of keywords. All searches used the MEDLINE collection/index.

MEDLINE Search #1: (((TS=(( ( cell OR mobile OR smartphone* OR smart OR portable ) NEAR/3 ( phone* OR device* OR app OR apps OR applicat* ) ) OR mhealth OR uhealth OR ehealth OR emental OR android OR iphone OR mobile OR app OR apps ))))) AND Language:(English) AND Document Types: (Classical Article OR Congresses OR Journal Article)

MEDLINE Search #2:

((((TS=((( mental OR psycholog* OR psychiatric OR emotional ) NEAR/3 ( health OR treatment* OR disorder* OR intervention* OR therapy OR distress OR affection ) ) OR depressi* OR anxiety OR "Ecological Momentary Intervention" ))))) AND Language: (English) AND Document Types: (Classical Article OR Congresses OR Journal Article)

MEDLINE Search #2:

(#1 AND #2) AND Language: (English) AND Document Types: (Classical Article OR Congresses OR Journal Article)

### APA PsycNET search

As proximity operators are not supported, we combined multiple “AND” and “OR” operators to partially reflect proximity operators. Obviously, we cannot fully emulate the semantics of the proximity operators using only boolean operators. PsycNEt does not support the option (in the query search user interface) for searching “Conference papers”, so we limited the search to “Journal Papers” and “Peer Reviewed Journals”.

*NOTE: the above search does not work directly in the search text box.*

(((((title: (mhealth))) OR ((Keywords: (mhealth))) OR ((abstract: (mhealth))) OR((title: (uhealth))) OR ((Keywords: (uhealth))) OR ((abstract: (uhealth))) OR ((title: (ehealth))) OR((Keywords: (ehealth))) OR ((abstract: (ehealth))) OR ((title: (emental))) OR ((Keywords: (emental))) OR ((abstract: (emental))) OR ((title: (android))) OR ((Keywords: (android))) OR((abstract: (android))) OR ((title: (iphone))) OR ((Keywords: (iphone))) OR ((abstract: (iphone)))OR ((title: (mobile))) OR ((Keywords: (mobile))) OR ((abstract: (mobile))) OR ((title: (app))) OR((Keywords: (app))) OR ((abstract: (app))) OR ((title: (apps))) OR ((Keywords: (apps))) OR((abstract: (apps)))) AND ((Year: [2013 TO 2018]) AND PublicationTypeFilt: ("Peer Reviewed Journal") AND ((DocumentType: ("Journal Article"))))) OR ((((((title: (cell)))) OR (((Keywords: (cell)))) OR (((abstract: (cell)))) OR (((title: (mobile)))) OR (((Keywords: (mobile)))) OR(((abstract: (mobile)))) OR (((title: (smartphone*)))) OR (((Keywords: (smartphone*)))) OR(((abstract: (smartphone*)))) OR (((title: (smart)))) OR (((Keywords: (smart)))) OR (((abstract: (smart)))) OR (((title: (portable)))) OR (((Keywords: (portable)))) OR (((abstract: (portable))))) AND((Year: [2013 TO 2018]) AND (PublicationTypeFilt: ("Peer Reviewed Journal")) AND(((DocumentType: ("Journal Article")))))) AND (((((title: (phone*)))) OR (((Keywords: (phone*))))OR (((abstract: (phone*)))) OR (((title: (device*)))) OR (((Keywords: (device*)))) OR (((abstract: (device*)))) OR (((title: (app)))) OR (((Keywords: (app)))) OR (((abstract: (app)))) OR (((title: (apps)))) OR (((Keywords: (apps)))) OR (((abstract: (apps)))) OR (((title: (applicat*)))) OR(((Keywords: (applicat*)))) OR (((abstract: (applicat*))))) AND ((Year: [2013 TO 2018]) AND(PublicationTypeFilt: ("Peer Reviewed Journal")) AND (((DocumentType: ("Journal Article"))))))))AND (((((((title: (mental)))) OR (((Keywords: (mental)))) OR (((abstract: (mental)))) OR (((title: (psycholog*)))) OR (((Keywords: (psycholog*)))) OR (((abstract: (psycholog*)))) OR (((title: (psychiatric)))) OR (((Keywords: (psychiatric)))) OR (((abstract: (psychiatric)))) OR (((title: (emotional)))) OR (((Keywords: (emotional)))) OR (((abstract: (emotional))))) AND ((Year: [2013 TO 2018]) AND (PublicationTypeFilt: ("Peer Reviewed Journal")) AND (((DocumentType: ("Journal Article")))))) AND (((((title: (health)))) OR (((Keywords: (health)))) OR (((abstract: (health)))) OR (((title: (treatment*)))) OR (((Keywords: (treatment*)))) OR (((abstract: (treatment*)))) OR (((title: (disorder*)))) OR (((Keywords: (disorder*)))) OR (((abstract: (disorder*)))) OR (((title: (intervention*)))) OR (((Keywords: (intervention*)))) OR (((abstract: (intervention*)))) OR (((title: (therapy)))) OR (((Keywords: (therapy)))) OR (((abstract: (therapy))))OR (((title: (distress)))) OR (((Keywords: (distress)))) OR (((abstract: (distress)))) OR (((title: (affection)))) OR (((Keywords: (affection)))) OR (((abstract: (affection))))) AND ((Year: [2013 TO 2018]) AND (PublicationTypeFilt: ("Peer Reviewed Journal")) AND (((DocumentType: ("Journal Article"))))))) OR ((((title: (depressi*))) OR ((Keywords: (depressi*))) OR ((abstract: (depressi*)))OR ((title: (anxiety))) OR ((Keywords: (anxiety))) OR ((abstract: (anxiety))) OR ((title: ("Ecological Momentary Intervention"))) OR ((Keywords: ("Ecological Momentary Intervention"))) OR((abstract: ("Ecological Momentary Intervention")))) AND ((Year: [2013 TO 2018]) AND PublicationTypeFilt: ("Peer Reviewed Journal") AND ((DocumentType: ("Journal Article"))))))AND Language: english
