# Supplementary material for: Smartphone Apps for the Treatment of Mental Disorders: Systematic Review
Source: JMIR Mhealth Uhealth. 2020 Apr 2;8(4):e14897. doi: 10.2196/14897 (PMC7163422; doi:10.2196/14897)
Supplement: Multimedia Appendix 3 [file mhealth_v8i4e14897_app3.docx]

## Supplementary Material C: Table 3. Key variables for ‘RCT’ or ‘pilot RCT’ studies.

**Table 3. Key variables (psychological approach, year, app name, mental disorder, study type, sample, experimental conditions and results) for ‘RCT’ or ‘pilot RCT’ studies.**

* *CBT*: Cognitive behavioral therapy; *3^rd^ WAVE*: 3rd wave therapy; *TRA*: Transdiagnostic therapy; *POS*: Positive Psychotherapy

* Type of assessment: *E*: Effect; *E and U/UX*= Effect and usability/user experience; *U/UX*= usability/user experience

| **Ref.** | **Psych. approach*** | **Year** | **Country** | **App**  **name** | **Mental disorder** | **Study design** | **Sample (N)** | **Experimental conditions** | **Main findings*** |
| --- | --- | --- | --- | --- | --- | --- | --- | --- | --- |
| [167] | CBT, 3^rd^ wave | 2013 | Australia | Get Happy Program | Depressive disorders | PILOT RCT | 35 | 1. Program using a mobile app (N=15)  2. Program using a computer (n=20) | E and U/UX:  - At post-treatment both the Mobile and Computer Groups were associated with statistically significantly benefits in depression.  - At 3 months follow-up the reduction seen for both groups remained significant.  -  54% of mobile app group and 64% of the computer group were very satisfied; 64% in both groups would be very confident in recommending this treatment to a friend. |
| [150] | CBT, POS | 2013 | Australia | myCompass | Various disorders | RCT | 720 | 1. My compass (app) (N=242)  2. Attention control (N=248)  3.Waiting list (N=230) | E and U/UX:  - At post-treatment myCompass group showed significantly greater improvement in symptoms of depression, anxiety, stress and in work and social functioning relative to both control conditions.  - At 3-month follow-up symptom scores remained at near normal levels.  - Participants in the attention control condition showed gradual symptom improvement during the post-intervention and their scores did not differ from the myCompass group at 3-month follow-up.  -  A high level of satisfaction with the myCompass app was reported without significant differences with attention control; 83% of myCompass participants reported that they would recommend the program to others. |
| [139] | CBT | 2013 | Italy | NA | Anxiety disorders | PILOT RCT | 25 | 1. Virtual Reality and Mobile Phone (N=8)  2. Biofeedback Virtual Reality and Mobile Phone  (N= 9)  3. Waiting list (N=8) | E and U/UX:  - The effectiveness of an immersive virtual relaxing environment may be improved by using physiological data to modify specific features of the virtual environment in real time.  - The mobile device was not only well tolerated but also appreciated by the patients. |
| [159] | CBT, 3^rd^ wave | 2014 | Sweden | NA | Depressive disorders | RCT | 81 | 1. Behavioral Activation intervention (N=40)  2. Mindfulness treatment (N=41) | E and U/UX:  - The two interventions did not differ significantly from one another.  - At 6-month follow-up for participants with  higher severity of depression the treatment based on BA was superior to the treatment based on mindfulness.  - At 6-month follow-up for participants with lower initial severity, the treatment based on mindfulness worked significantly better than the treatment based on BA.  -Participants in both groups rated their respective treatment as credible without significant differences between them |
| [55] | CBT | 2015 | United States of America | PRISM | Bipolar and related disorders | RCT | 82 | 1) Mobile device delivered interactive intervention linking patient-reported mood states with personalized self-management strategies (N=51)  2) Paper and pencil mood monitoring intervention (N=53 ) | E and U/UX:  -Conditions did not differ significantly in the impact on manic symptoms or functional impairment.  - Compared to the paper-and-pencil condition, participants in the augmented mobile intervention condition showed significantly greater reductions in depressive symptoms at 6 and 12 weeks.  - These effects were not maintained at 24-weeks follow up.  - Retention at 12 weeks was 93% and both groups were associated with high satisfaction. |
| [179] | CBT, 3^rd^ wave, POS | 2015 | United States of America | SuperBetter (SB) | Depressive disorders | RCT | 283 | 1. SB using cognitive-behavioral therapy and positive psychotherapy strategies to target depression (CBT-PPT SB) (N= 93)  2. SB version focused on self-esteem and acceptance (General SB) (N=97 )  3. Waiting list (WL) (N= 93) | E:  - At post-treatment and follow-up SB participants achieved greater reductions in depression scores than WL participants  - CBT-PPT SB did not perform better than General SB.  - Differences between SB versions favored General SB but were not statistically significant. |
| [188] | CBT, 3^rd^ wave | 2015 | Sweden | NA | Depressive disorders | RCT | 93 | 1. Blended treatment (four face-to-face sessions plus the smartphone application) (N=46)  2. Full-behaviour activation without the smartphone support (N=47) | E:  - Significant improvements in both groups across time the in depressive symptoms.  - The blended treatment reduced the therapist time with an average of 47%.  - The blended treatment approach could possibly treat nearly twice as many patients suffering from depression by using a smartphone application as add-on. |
| [188] | CBT | 2016 | United States of America | NA | Depressive disorders | RCT | 626 | 1. Cognitive intervention video game app (N= 209)  2. Problem solving therapy app (N= 211)  3. Information control (  provided daily health tips) app (N=206) | U/UX:  - Engagement: Study engagement was high during the first 2 weeks of treatment, falling to 44% adherence by the 4th week.  - Mobile randomized control trials can recruit large numbers of participants in a short period of time and with minimal cost. |
| [106] | CBT, 3^rd^ wave | 2016 | United States of America | PRIME | Schizophrenia spectrum and other psychotic disorders | RCT | 20 | 1. PRIME app (N=10)  2.Waiting-list treatment as usual control condition (N= 10) | U/UX:  - PRIME is a feasible and acceptable intervention for young people with schizophrenia.  - The overall satisfaction with PRIME was relatively high (100% study retention rate).  - Participants used the app, on average, every  other day, and were actively engaged with its various features each time they logged in. |
| [88] | CBT | 2016 | United States of America | PTSD Coach | Trauma and stressor-related disorders | PILOT RCT | 49 | 1. PTSD Coach (N=25)  2. Waiting list  (N=24) | E and U/UX:  - Between conditions effect size estimates were modest for PTSD symptom improvement, but not statistically significant.  - Participants reported that PTSD Coach components were moderately helpful and that they had learned tools and skills from the app to manage their symptoms.  - Participants reported using the app several times per week, throughout the day across multiple contexts, and endorsed few barriers to use. |
| [125] | 3^rd^ wave | 2016 | Sweden | Ångesthjälpen | Anxiety disorders | RCT | 152 | 1. Therapist-guided Internet-delivered Acceptance and Commitment Therapy (ACT)-based program and smartphone application (N=50)  2. Unguided Internet-delivered ACT-based program and smartphone application (N=51)  3. Waiting list (N=51) | E and U/UX:  - Treatment groups saw reduced general and social anxiety, but not panic symptoms compared to the waiting-list group.  - No differences in outcomes between guided and unguided interventions.  - Smartphone applications may partially compensate for lack of therapist support.  - 40% of the participants in the guided group and 29.4% of the participants in the unguided group completed all treatment and both groups did not differ in Internet platform usage |
| [172] | CBT, 3^rd^ wave, POS | 2016 | United States of America | MoodHacker | Depressive disorders | RCT | 300 | 1. MoodHacker intervention (N=150)  2. Alternative care consisting of links to vetted websites on depression (N=150) | E and U/UX:  - At 6-week follow-up, significant effects were found on depression, behavioral activation, negative thoughts, knowledge, work productivity, work absence, and workplace distress compared to alternative care subjects.  - MoodHacker yielded significant effects on depression symptoms, work productivity, work absence, and workplace distress for those who reported access to an employee assistance programs (EAP), but no significant effects on these outcome measures for those without EAP access.  - Significant effects on work absence in those with EAP access persisted at 10-week follow-up.  - Participants were mostly satisfied with the intervention and they reported good usability scores |
| [90] | CBT | 2016 | United States of America | PTSD Coach | Trauma and stressor-related disorders | PILOT RCT | 20 | 1. Self-Managed PTSD Coach (N=10)  2. Clinician-Supported PTSD Coach (N=10) | E and U/UX:  - Both treatments resulted in reductions in PTSD symptoms.  - Clinician Supported-PTSD Coach resulted in more specialty PTSD care use post intervention and possibly greater reductions in PTSD symptoms.  - Both PTSD Coach interventions are feasible and potentially helpful.  - The addition of clinician support appears to increase the effectiveness of self-management alone.  - Clinicians reported high satisfaction and ease of delivery and good veteran engagement across sessions and conditions.  - App usage among CS PTSD Coach participants was high. |
| [86] | CBT | 2017 | United States of America | PTSD Coach | Trauma and stressor-related disorders | RCT | 120 | 1. PTSD app Coach (N=62)  2. Waiting list ( N=58) | E:  - At post treatment, PTSD Coach participants  had significantly greater improvements in PTSD symptoms, depression symptoms, and psychosocial functioning than did waitlist participants  - There were no significant mean differences in outcomes between conditions.  - A greater proportion of PTSD Coach participants achieved clinically significant PTSD symptom improvement than waitlist participants. |
| [71] | CBT, POS | 2017 | United States of America | Virtual Hope Box (VHB) | Suicidal behavior disorder/nonsuicidal self-injury | RCT | 120 | 1. VHB app intervention group (N=58)  2. Control group that received printed materials about coping with suicidality (N=60). | E and U/UX:  - At 3 and 12 weeks VHB users reported significantly greater ability to cope  with unpleasant emotions and thoughts compared with the control group.  - No significant advantage was found on other outcome measures for treatment augmented by the VHB.  - Participants in the VHB group identifying the study intervention as helpful and were more likely to indicate an intention to use it again or to recommend it to someone else more than control condition. |
| [81] | CBT | 2017 | United States of America | Life Armor; PE Coach; Positive Activity Jackpot; Eventful; Tactical Breather; Virtual Hope Box; Daily Yoga; Simply Yoga | Trauma and stressor-related disorders | RCT | 144 | 1. Resilience enhancement group (N=72)  2. Control group: daily text messages for featuring positive aphorisms (N=72) | E:  - Both groups reported reductions in PTSD, anxiety, and depression symptoms during the 6-week intervention, which were sustained at 3 months, but exhibited partial rebound at 6–12 months.  - Smartphone apps can feasibly and effectively reduce symptom severity in subthres hold PTSD. |
| [70] | 3^rd^ WAVE, POS | 2017 | Australia | iBobbly | Suicidal behavior disorder/nonsuicidal self-injury | PILOT RCT | 61 | 1. Intervention (N=31)  2. Waiting list (N=30) | E:  - Pre intervention and post intervention changes on the suicidal ideation were significant in the ibobbly arm. These differences were not significant compared with the waitlist arm.  - ibobbly group showed statistically significant reductions in depression and anxiety scores compared with waitlist.  - No differences were observed in impulsivity. |
| [38] | CBT | 2017 | The Netherlands | Sleepcare | Sleep-wake disorders | RCT | 151 | 1. CBT-I delivered via the Sleepcare mobile phone app (N=74)  2. Waiting list (N=77) | E:  - The results showed significant interaction effects favoring the app condition on the primary outcome measures of insomnia severity and sleep efficiency.  - These improvements were also retained in a 3-month follow-up. |
| [184] | CBT | 2017 | Japan | Kokoro-App | Depressive disorders | RCT | 164 | 1. Antidepressants (N=83)  2. Antidepressants plus App (N=81) | E:  -Patients in the app group improved significantly more than control condition at week 9.  - The treatment benefits were maintained up to week 17. |
| [124] | CBT, TRA | 2017 | United Kingdom | Agoraphobia Free; Stress Free | Anxiety disorders | RCT | 170 | 1. A mobile app designed to target agoraphobia: Agoraphobia Free (N=86)  2. A mobile app designed to help with symptoms of anxiety in general: Stress Free (N=84) | E:  - Participants who received Agoraphobia Free did not improve more than those who received the Stress Free app.  - Both groups showed reductions in symptom severity over time that were statistically significant. |
| [79] | CBT | 2017 | United States of America | NA | Trauma and stressor-related disorders | RCT | 58 | 1. Anger management treatment (AMT) (N=37)  2. AMT augmented by a mobile application (app) system (N=37) | E and U/UX:  - Participants in both treatments demonstrated statistically significant and clinically meaningful reductions in anger severity and significant post-treatment reductions in PTSD.  - Veterans did not report significant changes in depression or interpersonal functioning.  -Participants reported the AMT plus the app as helpful and easy to use, |
| [182] | CBT | 2018 | Republic of Korea | TODAC TODAC (TT) | Depressive disorders | RCT | 34 | 1. Program TT app (N=17)  2. Control group (complete a daily mood diary) (N=17) | E:  - At post-treatment dysfunctional attitude scores in TT group were lower than they were in the control group. In clinical measures, both TT group and control group showed reduced in depression scores and anxiety at follow-up.  - TT group showed significantly reduced anxiety scores compared to control group. |
| [107] | CBT, 3^rd^ WAVE | 2018 | United States of America | PRIME | Schizophrenia spectrum and other psychotic disorders | RCT | 43 | 1. Intervention group (N=22)  2. Waiting list (N=21) | E and U/UX:  - People in the PRIME condition had significantly greater improvements in depression, defeatist beliefs, self-efficacy, and a trend towards motivation/pleasure negative symptoms post-trial compared to the WL  - These improvements were maintained 3 months follow up.  - PRIME condition had significantly greater improvements in components of social motivation post-trial.  - Participants rated their satisfaction with PRIME highly, which was demonstrated by the degree of engagement |
| [143] | CBT | 2018 | United States of America | FOCUS | Various disorders | RCT | 163 | 1. FOCUS app (N=82)  2. Wellness  Recovery Action Plan (WRAP) (N=81) | E and U/UX:  - Participants in both groups improved significantly in general psychopathology and depression without differences between them.  - Significant improvements in recovery were seen for the WRAP group post treatment.  - Significant improvements in recovery and quality of life were seen for the FOCUS group at six months.  - Satisfaction ratings were comparably high for both interventions. |
| [192] | CBT | 2018 | Germany-Switzerland-Norway | NA | Depressive disorders | RCT | 132 | 1.Psychological online interventions (POI) (N=65)  2. treatment as usual (N=67) | E and U/UX:  - At post-treatment, depression, behavioral activation, and dysfunctional attitudes did not differ between groups.  -Participants' evaluations reveal disappointment with the quality of the POI intervention and it did not meet their needs |
| [111] | CBT | 2018 | China | S-Health | Substance-related and addictive disorders | PILOT RCT | 75 | 1. Intervention group (N = 50)  2. Control group (N=25) | E and U/UX:  - At the end of the 1-month study trial, 26.2% of the intervention group and 50% of the control group had positive urine test results.  - The number of days using drug in the past week was significantly lower among participants in the intervention group relative to the control group  - The two groups did not differ in slopes (i.e., rates of change in outcomes measured weekly).  -Participants usability rates were good, without significant differences between them |
| [101] | CBT, 3^rd^ WAVE | 2018 | United Kingdom | Actissist | Schizophrenia spectrum and other psychotic disorders | RCT | 36 | 1. Actissist plus treatment as usual (TAU)  (N = 24)  2. ClinTouch (a symptom monitoring app) plus  TAU (N = 12) | E and U/UX:  - Treatment effects were large on negative symptoms, general psychotic symptoms and mood.  - The addition of Actissist conferred benefit at post-treatment assessment over routine symptom-monitoring and treatment as usual.  - Actissist was feasible and safe with high levels of user satisfaction. |
| [128] | CBT | 2018 | Sweden | Challenger | Anxiety disorders | RCT | 209 | 1. App and the self-help programme (parallel treatment) (N=70)  2. Self-help program for six  weeks and then the app for six weeks (sequential treatment) (N=70)  3. Waiting list (N=69) | E:  - No significant effect of adding the app to Internet-based selfhelp.  -Among participants actively using the app, adding Challenger to self-help resulted in significantly less social anxiety.  - At week 14, decreases in social anxiety were large for both the parallel and the sequential group with no differences between the active groups.  - Changes were maintained throughout the follow-up period. |
| [164] | CBT, 3^rd^ WAVE | 2018 | Germany | Be Good to Yourself | Depressive disorders | RCT | 90 | 1. Intervention group (N=45)  2. Waiting list (N=45) | E and U/UX:  - Trend level on the reduction of depressive symptoms in favor of the treatment group.  -Important to increase patients’ motivation to do it.  - The majority of participants evaluated the self-help application as positive and intended to use the application in the future. |
| [108] | CBT | 2018 | United Kingdom | Drink Less | Substance-related and addictive disorders | RCT | 672 | 1. Enhanced version of five app modules: Action Planning, Identity Change, Normative Feedback, Cognitive Bias Re-training, and Self-monitoring and feedback  (N=336)  2. Minimal version of the five app modules: (reduced functionality) versions  (N=336) | E and U/UX:  - There were no significant main effects of the  intervention modules on change in weekly alcohol consumption.  - After 4-weeks the combination of enhanced Normative Feedback and Cognitive Bias Re-training and enhanced Self-monitoring and feedback and Action Planning yielded improvements in alcohol outcomes.  - Enhanced Self-monitoring and Feedback was used significantly more often and rated significantly more positively for helpfulness, satisfaction and recommendation to others than the minimal version. |
| [69] | CBT, 3^rd^ WAVE, POS | 2018 | United States of America | BRITE | Suicidal behavior disorder/nonsuicidal self-injury | PILOT RCT | 66 | 1. Intervention As safe as possible (ASAP) supported by a smartphone app plus treatment as ususal (N=34)  2. Treatment as usual alone (N=32) | E and U/UX:  - The ASAP intervention did not have a statistically significant effect on suicide attempt, although results were in the hypothesized direction.  - Past history of a suicide attempt was a significant moderator of treatment outcome.  - There were no treatment effects on suicidal ideation.  - The majority of participants used the BRITE app.  - Participants reported high satisfaction with both the intervention and the app. |
| [141] | CBT | 2018 | Germany | NA | Anxiety disorders | RCT | 150 | 1. App intervention (N=60)  2. PC intervention (N=60)  3. Waiting list (N=30) | E and U/UX:  - At post-treatment both active conditions showed superior outcome on the composite of all social anxiety measures compared to the WL.  - No significant between-groups effects were found between the two active conditions.  - Treatment gains were maintained at 3-month follow-up.  - Program use was more evenly spread throughout the day in the mobile condition, indicating an integration of the program into daily routines. |
